# Right ventricle remodeling after transcatheter tricuspid leaflet repair in patients with functional tricuspid regurgitation: Lessons from the surgical experience

**DOI:** 10.3389/fcvm.2022.977142

**Published:** 2022-09-27

**Authors:** Alberto Albertini, Roberto Nerla, Fausto Castriota, Angelo Squeri

**Affiliations:** ^1^Cardiovascular Surgery Unit, Maria Cecilia Hospital GVM Care and Research, Cotignola, Italy; ^2^Interventional Cardiology Unit, Maria Cecilia Hospital GVM Care and Research, Cotignola, Italy; ^3^Cardiology Unit, Maria Cecilia Hospital GVM Care and Research, Cotignola, Italy

**Keywords:** tricuspid regurgitation, transcatheter tricuspid valve repair, right ventricular remodeling, right ventricular failure, cardiac magnetic resonance imaging

## Abstract

Clinically significant tricuspid regurgitation (TR) is common and associated with excess mortality. At the same time right ventricular (RV) failure is a complex clinical syndrome that results from many causes, but is often associated with long-term prognosis. Whilst results of isolated tricuspid valve (TV) surgery are often unsatisfactory and limited by the prohibitive risk of most patients, the recent development of percutaneous recovery techniques has opened new scenarios. In consideration of the complexity of the mechanisms that lead to right heart failure and RV dysfunction it is important to understand the real advantages that percutaneous TV treatment can offer, more specifically the effect of TR reduction on RV remodeling in the setting of functional tricuspid regurgitation (fTR).

## Introduction

Functional tricuspid regurgitation (fTR) is the most common cause of TR as it usually comes as a result of annular dilation and leaflets tethering caused by right ventricle (RV) enlargement and dysfunction due to pressure/volume overload related to left-sided heart disease (ventricular fTR) or atrial fibrillation (atrial fTR) (1, 2). It has become clear from surgical data of isolated tricuspid treatment that right ventricle (RV) end-systolic area, degree of RV dilatation, and presence of advanced heart failure can be predictors of poor outcomes (3, 4). On the other side, it has been recently demonstrated that patients showing early reverse remodeling of RV after tricuspid surgery were associated with a better long-term survival (5). Among the predictors of effective RV improvement in patients with pre-existing RV dysfunction, only right atrial pressure has been previously identified as associated with worse outcomes (5, 6).

As surgical repair tipically addresses annular dilatation, it is still unclear whether the same assumptions can be made for percutaneous treatments, in particular for those not directly acting on annulus size. In addition, scarce data is available about the effect of tricuspid treatment on RV function as assessed by advanced imaging techniques.

Aim of the present review is to evaluate the effect of percutaneous leaflet repair on RV function and size in the subgroup of patients with fTR.

## Percutaneous treatment of functional TR

FTR usually develops due to structural alterations in right atrial and/or ventricular myocardial geometry, leading to tricuspid annulus dilation and/or leaflet tethering, both associated with impaired leaflet coaptation. This is by far the most common cause of TR in adults, as shown by echocardiographic studies in which over than 90% patients with severe TR had a functional etiology (7, 8). Multiple etiologies of fTR have been described. Most commonly, fTR is associated with LV dysfunction and/or left-sided heart disease, leading to left atrial dilation, increased pulmonary wedge pressure and RV afterload (9, 10). Pulmonary hypertension arising from other causes than left heart disease, such as primary pulmonary hypertension, pulmonary embolism and chronic pulmonary disease, can also cause TR due to increased RV afterload, RV dilation and dysfunction (11). On the other hand, primary RV diseases, including isolated RV infarction or arrhythmogenic RV dysplasia, occur less frequently (12, 13). In addition, it should be acknowledged that RV dysfunction associated with significant TR is often difficult to assess (14). In all cases, anyway, when severe TR occurs, the increase in volume overload contributes to further RV dilation and dysfunction, with further malcoaptation of the TV leaflets perpetuating a vicious circle that impacts on prognosis. It is noteworthy, however, that RV reshaping process associated with fTR varies tremendously between patients (15). The different patterns of RV remodeling may be related to the underlying pathophysiology and to the timing in natural history of fTR when these patterns are assessed. We could consider two different pathophysiological groups:

1) patients without left-sided heart disease and without pulmonary hypertension, in whom significant fTR may appear attributable to right atrial dilation and atrial fibrillation, whereas the RV dimensions and function are usually within the normal values;
2) patients with left-sided heart disease and pulmonary hypertension, in whom TR is associated with various grades of RV dilation and dysfunction, usually related to myocyte loss and fibrosis that would finally affect prognosis.

While the benefit of tricuspid repair looks high in the former group, it is still not clear to what degree of RV dysfunction this process is reversible. In a study by Topilsky et al. (11), idiopathic TR was associated with basal RV enlargement (conical deformation) and tricuspid annulus dilation, whereas pulmonary hypertension–related TR was associated with increased RV length (elliptical deformation), causing tenting of the tricuspid leaflets and depicting a technically more challenging scenario to be solved with a percutaneous repair.

Tricuspid transcatheter edge-to-edge repair using the TriClip™ (Abbott Vascular, Santa Clara, CA, USA) or leaflet approximation with the PASCAL systems (Edwards Lifesciences, Irvine, CA, USA) are the currently available systems for percutaneous tricuspid repair. In the core lab-adjudicated TRILUMINATE study, percutaneous tricuspid repair in 85 prospectively enrolled patients (STR 84%; severe, massive and torrential in 29, 26 and 37%, respectively) was associated with a durable reduction to moderate TR or less in 71% at 1 year (16, 17). Interestingly, a significant reduction of RV and RA dimensions and improvement was reported in patients showing clinical and functional improvement. Of note, patients with severe RV dysfunction and severe pulmonary hypertension were excluded from the study and are currently not treated with percutaneous tricuspid repair. Favorable results have been obtained with the PASCAL system in a US-based early feasibility study (18); however, less experience has been accumulated and follow-up is shorter.

## Evaluation of right ventricular reverse remodeling

Evaluation of RV size and function is universally considered more complex than the one of left system. The biomechanics of the RV are significantly different from those of the LV and are optimized to deal with changes in volume load and to work at low pressure. This complex biomechanical construct and the change in relative components of radial function and longitudinal function with loading adds to the complexity in overall assessment of RV function (19).

Among all possible imaging modalities, echocardiography and cardiac magnetic resonance (CMR) should be considered as first-line tools to assess RV function changes over time (20, 21).

### Echocardiography

Echocardiography is by far the most common way to evaluate RV dimensions and function but technical issues are well known. Quantitative assessment of the RV by 2D transthoracic echocardiography is challenging due to its complex asymmetric geometry that leads to incomplete visualization in a single projection and lack of precise anatomic landmarks. Differently from LV, there are no geometrical assumptions for end-diastolic volume (EDV) and end-systolic volume (ESV) measurements. In addition, 2D echocardiography diameters of the RV may significantly vary just with minor rotation of the probe due to the lack of a precise and specific landmark, leading to a significant under- or over- estimation of RV size.

Evaluation of RV size and function is usually multiparametric and different echocardiographic techniques can be used. However, this usually leads to data fragmentation, thus preventing inter-study correlations. 3D echo can overcome these limitations but requires specific reconstruction software, it's time consuming and is limited by acoustic impedance.

3D echocardiography has demonstrated excellent correlation with CMR for the calculation of RV volumes and ejection fraction with a slight systematic underestimation of EDV and ESV but no difference in terms of RV ejection fraction (22).

### Cardiac magnetic resonance

CMR, even if limited by availability and costs, is considered the gold standard for evaluating RV volume, mass and function due to the volumetric acquisition providing excellent myocardial definition and tissue characterization. CMR permits direct EDV, ESV and EF measurement without geometrical assumption. Atrial arrhythmias might interfere with image acquisition and the presence of intrathoracic prosthetic material (prosthetic valves, pacemakers, etc.) in certain cases can lead to significant artifact making CMR images uninterpretable.

CMR not only allows precise evaluation of RV ventricular volumes and function but is able to calculate both left and right actual forward stroke and regurgitation volumes.

## Right ventricle remodeling after percutaneous tricuspid leaflet repair

Atrial fTR and ventricular fTR usually show two different remodeling patterns. The atrial fTR is linked with aging, atrial fibrillation, diastolic dysfunction, associated with a predominant atrial and annular dilatation with the latter as the predominant mechanism of TR. RV dilation in these cases is usually mild and limited to the basal segment.

Ventricular fTR has a different remodeling pattern with significant RV enlargement, less prominent annular dilatation and marked leaflets tethering as the leading mechanism of regurgitation. It is often due to left-sided ventricular or valve diseases, primary RV dysfunction as a consequence of RV infarction or cardiomyopathy, or pulmonary hypertension.

Residual TR and RV reverse remodeling after cardiac surgery are sensitive predictors of long-term prognosis (23) after surgery. In fact, successful reduction of TR after surgery can lead to a significant RV reverse remodeling by RV volume reduction and systolic function preservation, which translates into LV increase in preload and cardiac output.

Although available data is scarce, it seems that different degrees of reverse remodeling are linked to different etiologies of TR and degree of RV remodeling and dysfunction at the time of surgery. Several different papers showed a positive reverse remodeling after tricuspid surgical repair during mitral valve surgery (24), pulmonary endarterectomy or balloon pulmonary angioplasty (25, 26). On the contrary, there is some evidence that RV reverse remodeling is frequently absent in ischemic cardiomyopathy after CABG and mitral and tricuspid surgery (27).

**Figure 1:**
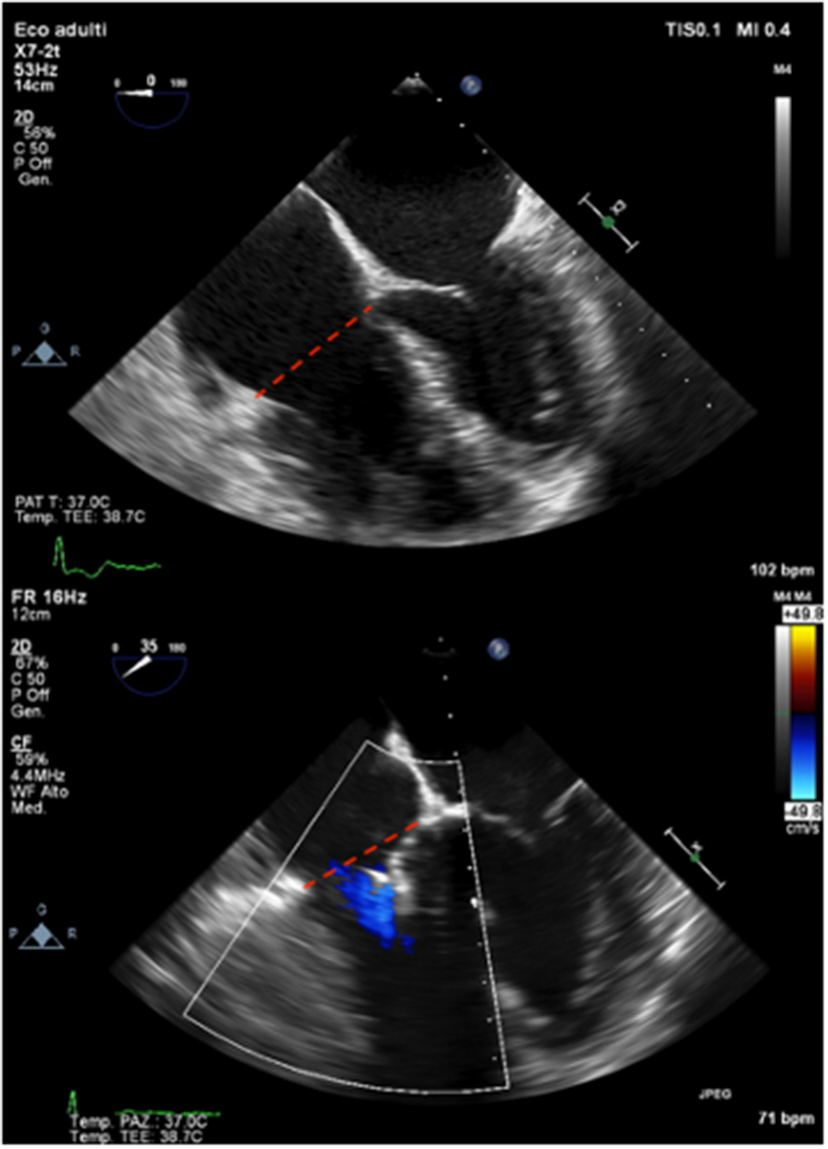
A case of RV reverse remodeling after percutaneous tricuspid leaflet repair. Baseline TEE (upper panel) and final result immediately after Triclip (bottom panel) showing early and significant reduction of TV annulus in end-diastole (red dashed lines).

**Figure 2:**
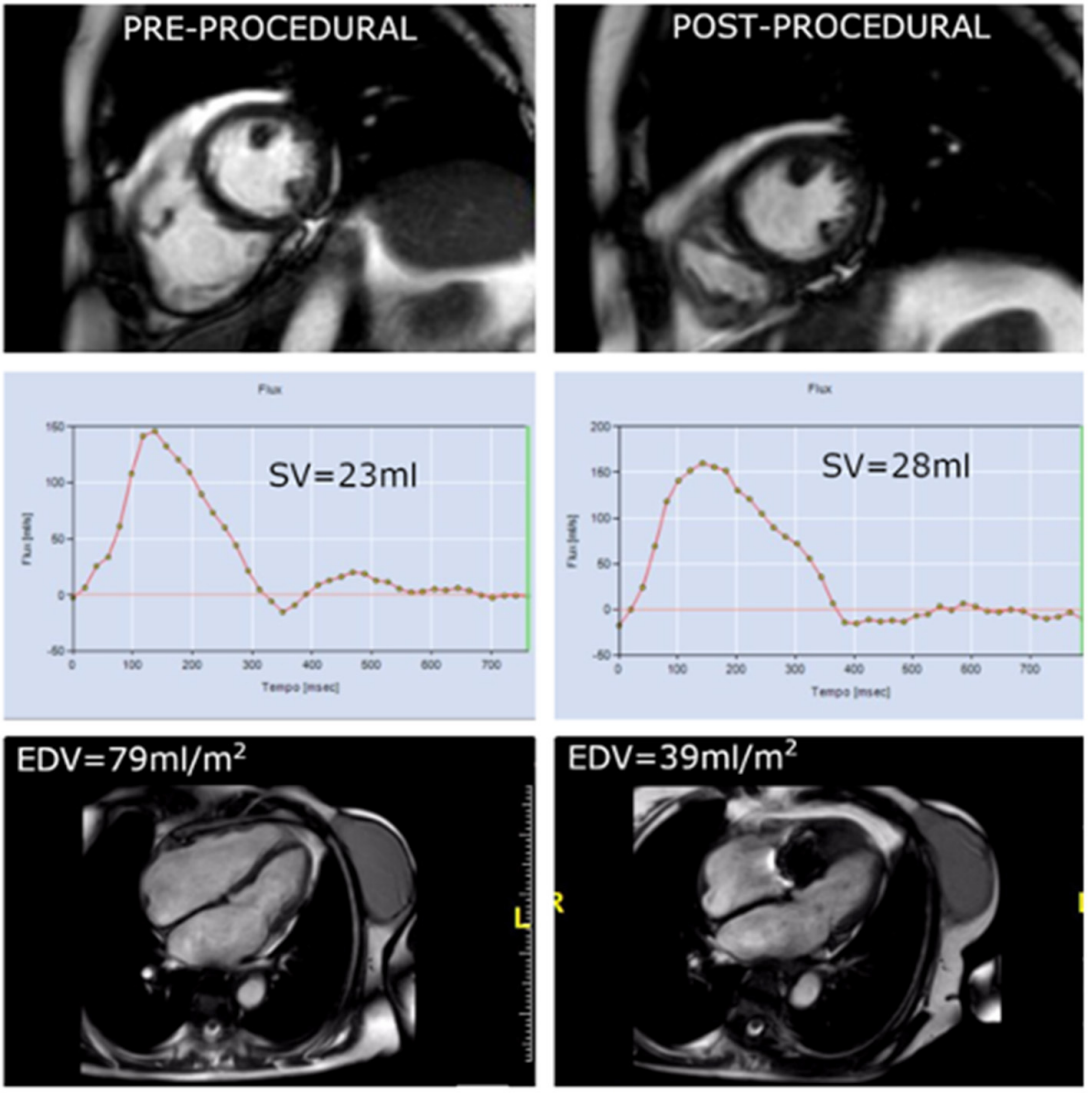
In the same patient showing early annulus size reduction, the comparison between pre-procedural CMR (*left column*) and immediately after procedure (*right column*) depicts a significant reduction in EDV as consequence of acute remodeling the day after the procedure. Phase contrast imaging shows a slight increase in absolute forward stroke due to reduction in regurgitant volume.

Surgical series should be considered as a reference guide to predict the effect of percutaneous repair on RV remodeling. Data derived from the Triluminate study (16) shows significant global and early RV reverse remodeling after successful procedure in terms of EDV, annular diameter, and atrial volumes at 30 days. These positive remodeling lasts till 1 year leading to an increase of RV contractility in terms of FAC and TAPSE. The study did not show if this positive reverse remodeling happens in all patients or in selected cases and it did not question whether it depends on the amount of TR reduction. RV reverse remodeling seems to happen early after the procedure, within 1 month (28) or even before discharge (29), with no further changes at 6 months or 1 year as a consequence of changed loading condition, as described by reduction in volumes without changes in contractility, as assessed by longitudinal strain (28). Reasons for the lack of RV reverse remodeling after 6 months are unclear and maybe due to incomplete TR reduction or irreversible structural changes in the RV myocardium at the time of Triclip intervention. Successful percutaneous tricuspid repair tends to reduce RV EDV with no significant changes in RVESV, which may be related to a significant reduction in RV volume overload with no significant reduction in RV afterload (30) (Figure 1). As a result, despite total RV stroke volume is reduced, forward RV stroke volume is significantly increased as documented by CMR studies (Figure 2) (28). The amount of reduction of RV end diastolic area, immediately after the procedure, is directly correlated with event-free survival at follow-up, confirming that RV reverse remodeling is an independent prognostic predictor (31).

Changes in RV loading condition and forward stroke volume lead to an improvement in left ventricular (LV) hemodynamic performance through an increase in LV preload documented by an increase of EDV and EF in the context of the so called “chronic latent LV under filling” (32). In this condition, a reduction of LV preload caused by TR can lead to an underestimation of mitral regurgitation. Moderate mitral regurgitation can become severe after tricuspid percutaneous repair as a consequence of an increase in RV forward stroke volume.

Finally, volume overload determined by severe TR is a major determinant of right atrium remodeling resulting in structural, electrical, or metabolic changes often leading to chronic atrial fibrillation (33). Irrespective of cardiac rhythm and RV loading conditions, right atrium volume is a major determinant of tricuspid annulus area in patients with FTR, and RA enlargement is an important mechanism for tricuspid annulus dilatation (34). Being able to reduce tricuspid annulus dimensions in the earlier phases of right atrium remodeling may be a promising target in preventing or delaying atrial fibrillation occurrence.

In conclusion, available preliminary data indicate an initial efficacy of percutaneous tricuspid repair on RV remodeling, with possible benefits to both ventricles. However, these results need confirmation from further and longer studies able to identify the subgroups of patients who may benefit more from the procedure.

## Author contributions

AA contributed to conception and design of the study. RN, AS, and FC wrote sections of the manuscript. All authors contributed to manuscript revision, read, and approved the submitted version.

## Conflict of interest

The authors declare that the research was conducted in the absence of any commercial or financial relationships that could be construed as a potential conflict of interest.

## Publisher's note

All claims expressed in this article are solely those of the authors and do not necessarily represent those of their affiliated organizations, or those of the publisher, the editors and the reviewers. Any product that may be evaluated in this article, or claim that may be made by its manufacturer, is not guaranteed or endorsed by the publisher.
